# Thrombosis and meningococcal infection rates in pegcetacoplan-treated patients with paroxysmal nocturnal hemoglobinuria in the clinical trial and postmarketing settings

**DOI:** 10.1016/j.rpth.2024.102416

**Published:** 2024-04-24

**Authors:** Richard J. Kelly, Hisakazu Nishimori, Regina Horneff, Peter Hillmen, Mohammed Al-Adhami, Stacie Lallier, Gloria F. Gerber

**Affiliations:** 1 St James’s University Hospital, Leeds, UK; 2 Department of Hematology, Okayama University Hospital, Okayama, Japan; 3 Hiroshima City Hiroshima Citizens Hospital, Hiroshima, Japan; 4 Swedish Orphan Biovitrum AB, Stockholm, Sweden; 5 Apellis Pharmaceuticals, Inc, Waltham, Massachusetts, USA; 6 Department of Medicine, Division of Hematology, Johns Hopkins University School of Medicine, Baltimore, Maryland, USA

**Keywords:** complement inhibition, paroxysmal nocturnal hemoglobinuria, pegcetacoplan, thrombosis, clinical trial, complement inhibitors, meningococcal infections, postmarketing drug surveillance

## Abstract

**Background:**

Paroxysmal nocturnal hemoglobinuria (PNH) is a rare, acquired hematologic disease characterized by complement-mediated hemolysis and thrombosis. Complement component 5 (C5) inhibitors have decreased PNH-related thrombosis rates and reduced mortality compared with those of age-matched controls. A small but significantly increased risk of life-threatening *Neisseria* infections, especially *N meningitidis*, represents a long-term safety risk of complement inhibition.

**Objectives:**

To evaluate the rates of thrombosis and meningococcal infections in patients with PNH treated with the complement component 3–targeted therapy pegcetacoplan.

**Methods:**

Cumulative patient-year exposure to pegcetacoplan was calculated, and thrombotic events and meningococcal infections were reviewed in 7 clinical trials and in the postmarketing setting. The clinical trial protocols and pegcetacoplan labeling required vaccination against *Streptococcus pneumoniae*, *N meningitidis*, and *Haemophilus influenzae* before pegcetacoplan use; the label allowed for prophylactic antibiotic use if pegcetacoplan must be administered before vaccination.

**Results:**

As of November 13, 2022, 464 patients with PNH had 619.4 patient-years of pegcetacoplan exposure in completed/ongoing clinical trials and the postmarketing setting. Seven thrombotic events were reported: 5 in clinical trials (2 in the same patient) and 2 in the postmarketing setting. The overall thrombosis rate was 1.13 events per 100 patient-years (clinical trials: 1.22 events/100 patient-years in 409.4 years; postmarketing: 0.95 events/100 patient-years in 210.0 years). No infections with meningococcal bacteria were reported.

**Conclusion:**

Event rates for thrombosis were comparable between pegcetacoplan and previously reported rates of C5 inhibitors in patients with PNH, and no cases of meningococcal infection were reported with pegcetacoplan. Continued follow-up is required.

## Essentials

• Terminal complement inhibition lowers thrombosis risk in paroxysmal nocturnal hemoglobinuria.
• We assessed thrombosis and meningitis rates with pegcetacoplan, a proximal complement inhibitor.
• Thrombosis rates on pegcetacoplan were comparable with those of terminal complement inhibitors.
• No meningitis infections were reported with pegcetacoplan, but continued surveillance is needed.

## Graphical abstract

**Figure:**
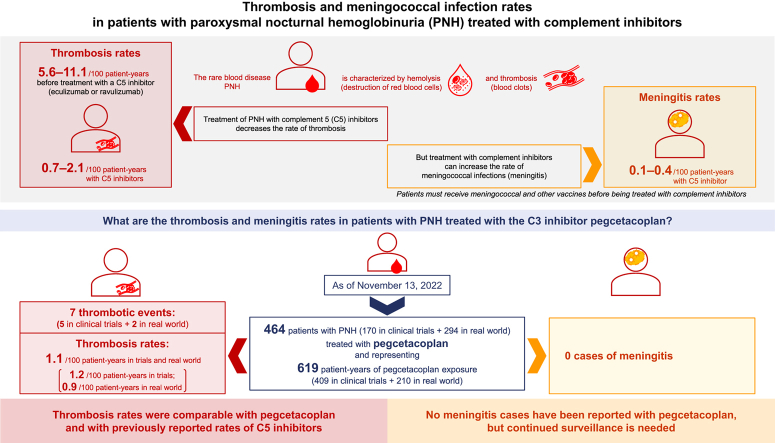

## 1 Introduction

Paroxysmal nocturnal hemoglobinuria (PNH) is a rare, acquired hematologic disease characterized by complement-mediated hemolysis and thrombosis [1]. Before complement inhibitor approval, thrombosis was the leading cause of death in PNH. Approximately 40% of patients experienced thrombotic events, which were fatal in almost 25% of cases [2,3]. Thrombosis mechanisms in PNH are likely multifactorial and may include the effects of intravascular hemolysis, complement-mediated leukocyte and platelet activation, inflammatory cytokines, and impaired fibrinolysis [4,5]. The approval and use of complement component 5 (C5) inhibitors changed the course of PNH and decreased thrombosis rates in patients with PNH, improving survival to comparable levels in age-matched controls [6]. In 195 patients who received the C5 inhibitor eculizumab in clinical trials between 2002 and 2005, thrombosis rates decreased from 7.37 events per 100 patient-years (1683 patient-years of exposure) without complement inhibition (ie, before eculizumab treatment) to 1.07 events per 100 patient-years (281 patient-years of exposure) with eculizumab [7]. The thrombosis rate with C5 inhibitor ravulizumab treatment was 1.21 events per 100 patient-years among 434 patients in the extension period (662 patient-years of exposure) [8].

Optimal PNH treatment must block complement activity enough to reduce the risk of thrombosis without compromising the complement system to an extent that would increase the risk of life-threatening infections, especially *Neisseria meningitidis* [9]. In a 10-year pharmacovigilance study of patients with PNH and atypical hemolytic uremic syndrome on eculizumab, the estimated meningococcal infection rate was 0.25 events per 100 patient-years (28,518 patient-years of global cumulative exposure to eculizumab) [10]. PNH-directed treatment must reduce thrombotic events and control hemolysis while considering the infection risk.

Pegcetacoplan is the first complement component 3 (C3)–targeted therapy approved in the United States for adults with PNH [11] and in Europe for adults with PNH who are anemic after receiving a C5 inhibitor for ≥3 months [12]. Clinical trial evidence has shown that pegcetacoplan improves hematologic parameters and anemia-related PNH complications, such as transfusion requirements, fatigue, and quality of life [[13], [14], [15]]. However, the long-term effects of pegcetacoplan on thrombosis and meningococcal infection risk have not been assessed. In this report, we evaluated the thrombosis and meningococcal infection rates among patients with PNH receiving pegcetacoplan in clinical trials and in real-world postmarketing settings.

## 2 Methods

This analysis included all patients who received ≥1 dose(s) of pegcetacoplan in 7 clinical trials and in the postmarketing setting in the United States, Europe, and the rest of the world as of November 13, 2022. Adults with PNH who completed 1 of 5 trials (PHAROAH [phase 1b, NCT02264639] [16], PALOMINO [phase 2a, NCT03593200] [17], PADDOCK [phase 1b, NCT02588833] [17], PEGASUS [phase 3, NCT03500549] [13,14], and PRINCE [phase 3, NCT04085601] [15]) could enroll in an ongoing, long-term, rollover, open-label, extension trial (phase 3, NCT03531255) [18]. Adolescents with PNH enrolled in the ongoing, phase 2, open-label PIONEER study (NCT04901936) [19] were also included. Adult patients (aged ≥18 years) in these studies were either anemic (hemoglobin of <10 g/dL [PHAROAH] [16] or <10.5 g/dL [PEGASUS] [13,14]) after receiving stable eculizumab dosing for ≥3 months or C5 inhibitor-naive (PALOMINO [17], PADDOCK [17], and PRINCE [15]); adolescent patients (aged 12-17 years) in PIONEER were anemic and were either C5 inhibitor-experienced or C5 inhibitor-naive [19]. In the United States, pegcetacoplan is available only through a Risk Evaluation and Mitigation Strategy program, which provided the numbers of patients who received pegcetacoplan in the US postmarketing setting. In other parts of the world, sales data were obtained from the manufacturer and distributor (Swedish Orphan Biovitrum AB), including the numbers of patients exposed to pegcetacoplan.

The clinical trial protocols and pegcetacoplan labeling required vaccination against *Streptococcus pneumoniae*, *N meningitidis* types A, C, W, Y, and B, and *Haemophilus influenzae* type B before pegcetacoplan initiation; if pegcetacoplan had to be administered before vaccination, the labels mandated prophylactic antibiotic use [[11], [12], [13], [14], [15], [16], [17]]. Patients receiving anticoagulant medications at trial entry were allowed to continue taking them with pegcetacoplan. All protocols were approved by the institutional review board or the independent ethics committee at each participating trial site, and patients provided informed consent for their participation. All trials were conducted in accordance with the protocols, applicable regulations, and ethical principles in the Declaration of Helsinki and the Good Clinical Practice guidelines.

Cumulative patient-years of pegcetacoplan exposure were calculated as the sum of the total duration of an individual patient’s exposure to pegcetacoplan in years. Postmarketing exposure duration was estimated assuming that each patient received 2 vials of pegcetacoplan per week (or 1 vial/3.5 d). Rates of thrombosis and meningococcal infection per 100 patient-years in clinical trials were based on the total number of events from the first pegcetacoplan dose to the last follow-up assessment. Thrombotic events (or history) in clinical trials were reported by system organ class and preferred term using the Medical Dictionary for Regulatory Activities High-Level Term of “pulmonary thrombotic and embolic conditions” and the Medical Dictionary for Regulatory Activities High-Level Group Term of “embolism and thrombosis,” which included arterial and venous thrombotic events. Postmarketing rates were estimated based on safety reports of total number of thrombotic (arterial and venous) and meningococcal infection events in the Apellis/Swedish Orphan Biovitrum AB global safety database, which included solicited reports from patient support and market research programs; spontaneous reports from health care providers, consumers, and regulatory agencies; and reports extracted from the literature. Compliance in the US postmarketing setting was calculated as the proportion of days a patient had the drug in possession divided by the total number of days of follow-up using central pharmacy prescription refill data following standard recommended practice [20,21]. Clinical trial data were used to describe the percentages of patients with ≥1 thrombotic event(s) before the trial initiation of pegcetacoplan therapy and the concomitant use of antithrombotic agents with pegcetacoplan therapy. Continuous data were summarized using descriptive statistics (ie, *n*, means, SDs, medians, and ranges). Categorical data were summarized using counts and percentages.

## 3 Results and Discussion

### 3.1 Patient characteristics

Baseline characteristics of adult patients with PNH who participated in the 5 completed pegcetacoplan trials are summarized in Table 1. Patients were recruited across multiple centers over the world and displayed demographic and clinical characteristics representative of the local trial center and recruiting criteria (ie, C5i-experienced vs C5i-naive). Among these patients, 22.6% (38/168) had ≥1 thrombotic event(s) before the trials; pegcetacoplan and antithrombotic treatments were received concurrently by 21.7% (35/161) of patients with available concomitant medication data.

**Table 1:** Baseline characteristics of adult patients with PNH enrolled in pegcetacoplan clinical trials.

Parent study name Characteristic	PHAROAH (*n* = 9)	PALOMINO (*n* = 4)	PADDOCK (*n* = 22)	PEGASUS (*n* = 80)	PRINCE (*n* = 53)
Age (y), mean (range)	46.0 (24, 72)	30.8 (22, 47)	42.2 (22, 67)	48.8 (19, 81)	44.5 (20, 74)
Female, *n* (%)	8 (88.9)	3 (75.0)	9 (40.9)	49 (61.3)	24 (45.3)
Race, *n* (%)					
American Indian or Alaska Native	0	0	0	0	11 (20.8)
Asian	0	0	15 (68.2)	12 (15.0)	39 (73.6)
Native Hawaiian/other pacific islander	0	0	1 (4.5)	0	0
Black	1 (11.1)	0	0	2 (2.5)	2 (3.8)
White	8 (88.9)	4 (100.0)	3 (13.6)	49 (61.3)	0
Others	0	0	3 (13.6)	1 (1.3)	1 (1.9)
Not reported	0	0	0	16 (20.0)	0
Ethnicity, *n* (%)					
Hispanic or Latino	1 (11.1)	0	0	3 (3.8)	14 (26.4)
Not Hispanic or Latino	8 (88.9)	4 (100.0)	21 (95.5)	61 (76.3)	39 (73.6)
Unknown	0	0	1 (4.5)	16 (20.0)	0
Body mass index (kg/m^2^), mean (SD)	34.0 (12.2)	27.2 (5.2)	25.0 (4.5)	26.3 (4.3)	23.7 (4.0)
Time since PNH diagnosis (y), median (range)	9.0 (1, 22)	1.8 (0.8, 7.0)	9.2 (1.1, 38.8)	6.5 (1.0, 38.0)	3.8 (0.1, 27.0)
No. of packed red blood cell transfusions received in year prior to trial entry, mean (SD)	5.6 (4.5)	5.0 (2.9)	5.0 (4.5)	6.5 (7.5)	4.3 (4.6)
Thrombosis history, *n* (%)	3 (33.3)	3 (75.0)	2 (9.1)	25 (31.3)	5 (9.4)
Hemoglobin (g/dL), mean (SD)	9.2 (1.1)	7.7 (0.9)	8.5 (1.8)	8.7 (1.0)	9.1 (1.3)
Lactate dehydrogenase (U/L), mean (SD)	Not reported	2548.8 (631.1)	2354.9 (988.0)	282.4 (211.0)	2081.3 (937.9)
Reticulocyte count, 10^9^ cells/L, mean (SD)	Not reported	238.3 (91.0)	198.2 (63.0)	216.9 (71.7)	213.2 (93.5)
Prior antithrombotic therapy, *n* (%)	2 (22.2)	3 (75.0)	8 (36.4)	27 (33.8)	11 (20.8)
Concomitant antithrombotic therapy, *n* (%)	2 (22.2)	0	10 (45.5)	20 (25.0)^a^	3 (5.7)

PNH, paroxysmal nocturnal hemoglobinuria.

a Taken only during pegcetacoplan monotherapy in the 16-week randomized controlled period and the 32-week open-label period in PEGASUS.

### 3.2 Overall pegcetacoplan exposure and compliance

Overall, 464 patients with PNH had a total of 619.4 patient-years of pegcetacoplan exposure in completed and ongoing clinical trials (170 patients and 409.4 patient-years of exposure) and in the postmarketing setting, including the United States, Europe, and the rest of the world (294 patients and 210.0 patient-years of exposure) as of November 13, 2022 (Table 2). Compliance in the US postmarketing setting was estimated to be 98% as of November 13, 2022.

**Table 2:** Pegcetacoplan exposure, thrombosis, and meningococcal infections in patients with paroxysmal nocturnal hemoglobinuria.^a^

Exposure and events of interest	PHAROAH^b^	PALOMINO^b^	PADDOCK^b^	PIONEER	PEGASUS^b^	PRINCE^b^	All clinical trials	Postmarketing setting^c^	Total
Received ≥1 dose(s), *n*	9	4	22	3	80	52	170	294	464
Cumulative exposure, patient-years	20.3	15.8	65.0	3.5	188.3	116.4	409.4^d^	210.0	619.4
Thrombotic events, *n*	1	0	0	0	4^e^	0	5	2	7
Meningococcal infections, *n*	0	0	0	0	0	0	0	0	0

a As of November 13, 2022.

b Exposure and events were assessed during the completed clinical trial throughout the ongoing, long-term, rollover, open-label, and extension trial.

c Exposure and events reported through pharmacovigilance and postmarketing distribution programs in the United States, Europe, and the rest of the world.

d Rounded number.

e One patient experienced 2 events.

### 3.3 Incidence of thrombosis

A total of 7 thrombotic events were reported: 5 in clinical trials (2 in the same patient) and 2 in the postmarketing setting (Table 2). Of the 5 thrombotic events reported in the clinical trials, 1 patient in PHAROAH experienced an event of portal vein thrombosis in the setting of likely breakthrough hemolysis and sepsis that developed after placement of a kidney stent and a nephrostomy tube. Three patients in PEGASUS experienced events: 1 patient in the pegcetacoplan-to-pegcetacoplan group had a deep vein thrombosis associated with a peripherally inserted central catheter line in the setting of diffuse large B cell lymphoma during the 48-week, open-label extension period; 1 patient in the eculizumab-to-pegcetacoplan group had a jugular vein thrombosis (presumably associated with central catheter placement) in the setting of multiple complications post cholecystectomy after switching from eculizumab to pegcetacoplan during the 48-week, open-label extension period; and 1 patient in the pegcetacoplan-to-pegcetacoplan group experienced 2 events (deep vein thrombosis and venous embolism) during the long-term, open-label extension trial within 3 weeks of discontinuing pegcetacoplan and switching to eculizumab in order to conceive (Table 3). None of these events were assessed as related to pegcetacoplan by study investigators, and all were resolved.

**Table 3:** Thrombotic events reported in pegcetacoplan clinical trials.^a^

Patient sex, age (y), trial	Event Type, severity^b^, seriousness, duration	Laboratory values Hb (g/dL)^c^ LDH (U/L)^d^	Peri-event antithrombotic therapy	Narrative
Female, 36, PHAROAH	Portal vein thrombosis, severe, serious, days 207-229	• Day 207 Hb = 8.7^e^ • Day 221 Hb = 6.4^e^ • Day 229 Hb = 8.7	Enoxaparin sodium (days 207-229)	• History of lower-extremity DVT, aplastic anemia, diabetes, and multiple sclerosis • Developed sepsis after placement of a kidney stent and a nephrostomy tube for planned left staghorn calculus removal (day 199) • Received concomitant antithrombotic treatment (rivaroxaban) before the event
Male, 66, PEGASUS	DVT (PICC-line–associated), severe, serious, days 285-291	• Day 268 Hb = 8.6^f^; LDH = 1228^g^ • Day 295 Hb = 10.7^f^	Enoxaparin sodium for VTE prophylaxis (days 268, 271-273, 275-280, and 282-283) and tinzaparin sodium (days 286-310)	• History of aplastic anemia • No history of thromboembolic events • New diagnosis of diffuse large B cell lymphoma (day 278) • PICC line inserted because of poor venous access to begin R-CHOP chemotherapy (day 284)
Male, 35, PEGASUS	Jugular vein thrombosis, moderate, nonserious, days 169-169^h^	• Day 149 LDH = 3000^e^ • Day 170 Hb = 6.0; LDH = 438	Heparin (days 154-169, or longer)	• No relevant history, including no history of thromboembolic events • Multiple complications post cholecystectomy, including acute respiratory failure due to sepsis (day 149), circulatory collapse (day 149), and acute renal failure requiring hemodialysis (day 154) • Received concomitant antithrombotic treatment (tinzaparin sodium) before the event
Female, 35, PEGASUS	DVT, mild, nonserious, subclavian vein TE, moderate, nonserious, days 569-603	• Day 568 Hb = 6.0	Acetylsalicylic acid (days 569-599) and enoxaparin sodium (days 570-592)	• History of thrombocytopenia • No history of thromboembolic events • Discontinued pegcetacoplan to conceive (day 552) • Received eculizumab 1200 mg once every 2 weeks (days 554-582) • Received concomitant antithrombotic treatment (heparin as needed for port placement) with pegcetacoplan before the event

DVT, deep vein thrombosis; Hb, hemoglobin; LDH, lactate dehydrogenase; PICC, peripherally inserted central catheter; R-CHOP, rituximab-cyclophosphamide-doxorubicin-vincristine (Oncovin)-prednisone regimen; TE, thromboembolism; VTE, venous thromboembolism.

a Additional details of the postmarketing findings are not available at this time.

b Severity was quantified by study investigators.

c Hb reference range is 12.0 to 16.9 g/dL for females and 13.6 to 18.0 g/dL for males unless noted otherwise.

d LDH reference range is 113 to 226 U/L unless noted otherwise.

e Reference ranges not specified.

f Hb reference range is 13.5 to 18.0 g/dL.

g LDH reference range is 120 to 246 U/L.

h Patient later experienced a severe, serious event of mesenteric ischemia possibly related to pegcetacoplan (per investigator’s assessment) on study days 220 to 233, which led to pegcetacoplan and study discontinuation. Patient recovered from intestinal ischemia on day 233.

The calculated overall thrombosis rate was 1.13 events per 100 patient-years, with 1.22 events per 100 patient-years in 409.4 years of pegcetacoplan exposure in clinical trials and 0.95 events per 100 patient-years in 210.0 years of postmarketing pegcetacoplan exposure. One event of severe mesenteric ischemia in the eculizumab-to-pegcetacoplan group that was possibly related to pegcetacoplan and could have been due to thrombosis in the PEGASUS 48-week, open-label extension was not included [14]. Some patients had a history of prior thrombosis (22.6%) and/or were concomitantly receiving antithrombotic agents (21.7%) with pegcetacoplan, which might have influenced patients’ risk for thrombosis during the trials. Of the 4 patients who experienced a thrombotic event, 1 had a prior history of thrombosis and 3 were receiving concomitant antithrombotic therapy (Table 3).

Previous studies have shown decreased thrombosis rates with C5 inhibitors compared with those with supportive care without complement inhibitors. In 79 eculizumab-treated patients at a single center from May 2002 to July 2010, the thrombotic event rate decreased from 5.6 events per 100 patient-years before treatment to 0.8 events per 100 patient-years during treatment [6]. A pooled analysis from the 3 clinical trials and the extension study of eculizumab showed that the thromboembolism rate during eculizumab treatment was reduced by 85% from a pretreatment rate of 7.37 events per 100 patient-years to 1.07 events per 100 patient-years [7]. A long-term safety analysis of 195 patients receiving eculizumab over 66 months reported an 82% reduction in the thromboembolism rate, from 11.13 events per 100 patient-years before treatment to 2.14 events per 100 patient-years during treatment (10 events/467.1 patient-years of exposure) [22]. In a 2-year extension of 2 ravulizumab clinical trials, the thrombotic event rate was 1.21 events per 100 patient-years (8 events in 434 patients with 662 patient-years of exposure) [8]. In a recent report of 509 patients with PNH receiving C5 inhibitors from May 2002 to July 2022 in the United Kingdom, 23 patients had a thrombotic event, consistent with a thrombosis rate of 0.73 events per 100 patient-years [23].

### 3.4 Incidence of meningococcal infections

No cases of meningococcal infection occurred during any of the completed or ongoing pegcetacoplan clinical trials or in the postmarketing setting through November 13, 2022 (Table 2). By comparison, no meningococcal infections were reported in the 2-year extension study of ravulizumab (662 patient-years) [8], but 1 fatal case and 1 life-threatening case of meningococcal sepsis were reported in patients receiving ravulizumab for 2.2 years and 1 year, respectively [24,25]. Several cases of encapsulated bacterial infections, including meningococcal infections, have also been reported in patients receiving eculizumab [26,27]. In a recent international PNH registry study of patients with PNH who received meningococcal vaccination before being treated with eculizumab and were receiving prophylactic antibiotics during C5 inhibitor treatment, the meningococcal infection rate was 0.1 per 100 (95% CI, 0.0-0.4) patient-years [28]. A pharmacovigilance analysis that evaluated eculizumab use in patients with PNH and atypical hemolytic uremic syndrome over 10 years found a meningococcal infection rate for patients with PNH of 0.25 per 100 patient-years (approximately 1000-2000 times higher than that of the general population), with a fatal meningococcal infection rate of 0.03 per 100 patient-years [10]. However, in the recent UK study of 509 patients with PNH, 11 cases of *Neisseria meningitis* were reported in 10 patients over 3130 years of C5 inhibitor treatment, representing a rate of 0.35 events per 100 patient-years [23]. Ongoing surveillance is needed to determine if the absence of meningococcal infections in pegcetacoplan-treated patients is due to the treatment itself or another cause, such as differences in vaccination availability within the patient populations. Awareness of meningococcal risk in patients with PNH receiving complement inhibitors, especially with rare forms not covered by vaccination, remains warranted [23,25].

### 3.5 Limitations

Study limitations include a reliance on voluntary adverse event reporting in the postmarketing setting, which may have underestimated event occurrence. This analysis included adolescents, whereas the thrombosis rates reported from the eculizumab and ravulizumab trials included adults only, although data from only 3 adolescents (PIONEER) are not expected to significantly influence the results. The effect of differing inclusion criteria between the pegcetacoplan and the C5 inhibitor clinical trials on event rates cannot be discounted; the overall percentage of patients with a history of thrombosis is comparable, although patients with history of stroke or myocardial infarction were excluded from the PEGASUS trial. Given the time to reach pharmacologic steady state with subcutaneous infusion, further study of pegcetacoplan monotherapy is necessary in patients who present with an acute PNH-related thrombotic event. Severe intravascular hemolysis is associated with thrombosis, and additional studies of thrombotic risk in patients with PNH experiencing breakthrough hemolysis are required [29]. The influence of individual risk factors, such as prior history of thrombosis and concomitant antithrombotic agents on likelihood of thrombosis, or immunization status and prophylactic antibacterial therapy on meningococcal infection, could not be evaluated in this analysis; results from such assessments could help clinicians determine the most appropriate therapeutic approach for individual patients.

## 4 Conclusions

Thrombotic event rates were comparable between the C3-targeted therapy pegcetacoplan and the C5 inhibitors eculizumab and ravulizumab in patients with PNH. No cases of meningococcal infection were reported after 619.4 patient-years of pegcetacoplan exposure. Thus far, proximal complement inhibition with pegcetacoplan has not displayed a significant increase in meningococcal infections compared with C5 inhibitors; however, definitive conclusions are limited given the low event rate. Continued follow-up is required to understand the long-term risks associated with C3 inhibition in patients with PNH.
